# Evaluación del papel de diversos biomarcadores en el desarrollo de eventos cardiovasculares adversos mayores en pacientes sometidos a cirugía cardíaca

**DOI:** 10.1515/almed-2020-0106

**Published:** 2020-12-10

**Authors:** Claudia E. Imperiali, Juan C. Lopez-Delgado, Macarena Dastis-Arias, Lourdes Sanchez-Navarro

**Affiliations:** Laboratorio Clínico, Hospital Universitari de Bellvitge, L’Hospitalet de Llobregat, Barcelona, Spain; Critical Care Unit, Hospital Universitari de Bellvitge, L’Hospitalet de Llobregat, Barcelona, Spain; Clinical Laboratory, Hospital Universitari de Bellvitge, L’Hospitalet de Llobregat, Barcelona, Spain

**Keywords:** biomarcadores, cirugía cardíaca, pronóstico

## Abstract

**Objetivos:**

Aunque los eventos cardiovasculares adversos mayores (MACE) son frecuentes en el postoperatorio de la cirugía cardíaca (CC), no se suele evaluar el riesgo de desarrollarlos. Analizamos una serie de biomarcadores postoperatorios potencialmente relacionados con el desarrollo de MACE durante el postoperatorio de CC.

**Métodos:**

Se incluyeron 210 pacientes de CC. Se consideraron MACE el infarto agudo de miocardio, fallo cardíaco, ictus durante el ingreso en la unidad de cuidados intensivos, y mortalidad a los 30 días tras la CC. Se midieron la troponina T de alta sensibilidad (hs-TnT), proteína C reactiva (PCR), procalcitonina (PCT), interleucina 6 (IL-6) en plasma y la fracción de plaquetas inmaduras (IPF) en sangre al ingreso en la UCI y a las 24 h. Se calculó la diferencia entre ambas medidas (Δ) para evaluar la relación entre estos biomarcadores y MACE. Los pacientes con infección inmediata tras la CC (n=13) fueron excluidos del análisis final.

**Resultados:**

Las intervenciones más frecuentes fueron la cirugía univalvular (n=83; 38%) y la cirugía de revascularización coronaria (n=72; 34%). Se diagnosticaron MACE postoperatorios en 31 (14.8%) pacientes. Los pacientes con MACE mostraron una elevación de biomarcadores a las 24 h con respecto al ingreso en la UCI. Se observó una relación independiente entre ΔIPF (OR: 1.47; 95% CI: 1.110–1.960; p=0.008) y Δhs-TnT (OR: 1.001; 95% CI: 1.0002–1.001; p=0.008) y los MACE.

**Conclusiones:**

Las concentraciones postoperatorias de ΔIPF y Δhs-TnT pueden ser útiles para identificar a pacientes con riesgo de desarrollar MACE.

## Introducción

La cirugía cardíaca (CC) es un tipo de cirugía mayor con una elevada morbi-mortalidad [1]. A pesar de los avances en anestesiología, los procedimientos quirúrgicos y los cuidados postoperatorios, las complicaciones cardiovasculares siguen siendo una importante causa de morbi-mortalidad postoperatoria [2]. De este modo, el desarrollo de eventos cardiovasculares adversos mayores (MACE) no es infrecuente en los pacientes que se han sometido a cirugía mayor [3]. Los MACE incluyen eventos cardiovasculares como el infarto agudo de miocardio, fallo cardíaco, ictus y mortalidad postoperatoria [4].

Para evaluar el riesgo de MACE en los pacientes quirúrgicos, se han tenido en cuenta diferentes factores de riesgo como la edad, sexo, y la presencia de comorbilidades. Sin embargo, el desarrollo de MACE no solo está asociado a la presencia de estos factores de riesgo o de otras comorbilidades, sino que diversos factores intraoperatorios pueden influir en el desarrollo de complicaciones postoperatorias y, en consecuencia, de MACE [5]. La respuesta inflamatoria que se produce tras un procedimiento quirúrgico intenso y un *bypass* cardiopulmonar prolongado podría ser crucial en el desarrollo de eventos adversos cardiovasculares tras la CC, lo cual subraya la relación entre la inflamación y el pronóstico [6], [7]. Algunos biomarcadores podrían desempeñar un papel fundamental a la hora de predecir el desarrollo de MACE. Así, los biomarcadores cardíacos como la troponina I o T de alta sensibilidad (hs-TnI/T) han mostrado ser efectivos a la hora de predecir el desarrollo de eventos cardiovasculares adversos [8]. Por otro lado, no existe una relación clara entre algunos biomarcardores inflamatorios como la proteína C reactiva (PCR), la procalcitonina (PCT), y la interleucina-6 (IL-6) y MACE, aunque parece existir una fuerte relación [9], [10], [11], [12], [13].

Recientemente, se han incrementado los estudios que ponen de manifiesto el papel de la fracción de plaquetas inmaduras (IPF), también llamadas plaquetas reticuladas, como un posible factor predictivo de MACE después de la cirugía [5], [14], [15]. Las plaquetas reticuladas son metabólica y enzimáticamente más activas que las plaquetas maduras, y tienen mayor actividad protrombótica, la cual está estrechamente relacionada con el estado inflamatorio descompensado [16]. Además, recientemente se ha demostrado que la IPF se asocia a la respuesta inflamatoria que, finalmente, determinan el resultado de la CC [17]. En consecuencia, parece probable que la IPF esté relacionada con MACE tras la CC. La utilidad de la IPF se basa en el hecho de que ésta se puede medir fácilmente junto a la realización del hemograma.

Atendiendo a estos datos, postulamos como hipótesis que los biomarcadores cardíacos e inflamatorios podrían estar relacionados con el desarrollo de eventos cardiovasculares y la mortalidad tras la CC. Por tanto, el objetivo de este estudio es evaluar la relación entre una serie de biomarcadores postoperatorios como la hs-TnT, PRC, PCT, IL-6, y la IPF y el desarrollo de MACE en el contexto de la CC.

## Materiales y métodos

### Pacientes

Este estudio prospectivo, observacional, unicéntrico se llevó a cabo en un hospital universitario de tercer nivel entre marzo de 2016 y marzo de 2017. El estudio recibió la aprobación del Comité de Ética Institucional correspondiente (Referencia Nº PR090/16). Todos los pacientes firmaron un consentimiento informado con anterioridad a su inclusión en el estudio. Este estudio se realizó de conformidad con los principios de la Declaración de Helsinki y las buenas prácticas clínicas.

Los criterios de inclusión fueron tener 18 años o más y haberse sometido a CC con un ingreso en la Unidad de Cuidados Intensivos (UCI) durante al menos 48 horas. Por CC se entienden las siguientes intervenciones: cirugía de revascularización coronaria (CABG), cirugía valvular, cirugía aórtica y resección de tumores cardíacos. Los criterios de exclusión fueron: inmunosupresión previa, enfermedades hematológicas y oncológicas en los últimos cinco años y trasplante coronario.

Todas las intervenciones desarrolladas durante el periodo de estudio fueron realizadas por el mismo grupo de cirujanos cardíacos mediante esternotomía media, *bypass* cardiopulmonar (CEC) estándar con hipotermia moderada (>34 °C) y cardioplejía anterógrada. Durante la CC, se mantuvo una presión aórtica media de >65 mmHg. Para la revascularización, se emplearon injertos de la arteria torácica interna (o bilateral, en caso de ser posible) y de la vena safena. El flujo del injerto del *bypass* se comprobó mediante flujometría Doppler por tiempo de tránsito. Todos los pacientes fueron trasladadosa la UCI tras la cirugía. Siguiendo el protocolo del hospital, se administró tratamiento antibiótico profiláctico a la totalidad de los pacientes tras la cirugía. En todos los pacientes, el manejo postoperatorio en la UCI fue realizado por el clínico responsable siguiendo los protocolos del hospital.

Los datos se extrajeron del historial médico electrónico de los pacientes y se incorporaron a una base de datos para su posterior análisis. De forma sistemática, se recogieron los datos preoperatorios (esto es, datos demográficos, comorbilidades y tratamiento previo a la cirugía), datos operatorios y variables postoperatorias.

### Materiales y métodos

La principal variable a estudiar fue la presencia de MACE durante el periodo de seguimiento. El desarrollo de MACE se registró ante la presencia de alguna de las complicaciones descritas tras el ingreso en la UCI, inmediatamente después de la cirugía, hasta el alta de la UCI. También se registró la mortalidad a los 30 días tras la CC. El diagnóstico de MACE se confirmó por la presencia de al menos una de las siguientes complicaciones: infarto agudo de miocardio, fallo cardíaco, ictus durante el ingreso en la unidad de cuidados intensivos, y mortalidad por cualquier causa. El infarto agudo de miocardio, fallo cardíaco, y el ictus se definieron siguiendo las definiciones actuales y la práctica clínica [18], [19], [20], [21], [22], [23], [24]. El infarto agudo de miocardio se definió como la presencia de una repolarización anormal en el electrocardiograma (esto es, ondas Q, elevación o descenso del segmento ST y bloqueo de rama izquierda), evidencia en las pruebas de imagen de nuevas anomalías en el movimiento parietal regional y la elevación de las concentraciones de biomarcadores cardíacos en plasma [18], [19], [20], [21], [22], [23], [24]. El ictus se definió como la presencia de un episodio agudo de déficit neurológico global o focal [21], [22]. El fallo cardíaco se definió como la aparición reciente de deterioro o empeoramiento de la función cardíaca en pacientes con fallo cardíaco previo, y la necesidad de administrar inotrópicos o vasopresores, para mantener la estabilidad hemodinámica durante el periodo postoperatorio [19], [23]. Se realizó una ecocardiografía o vigilancia invasiva en los casos más graves (p.ej. cateterización de la arteria pulmonar) para confirmar el diagnóstico de fallo cardíaco postoperatorio [24].

### Extracción de sangre y medición de biomarcadores

Las muestras de sangre se extrajeron mediante un catéter venoso central al ingreso en la UCI inmediatamente tras la cirugía y a las 24 horas. El plasma se almacenó en tubos de 4 mL con heparina de litio (Vaccuette^®^, Greiner Bio One^®^, Kremsmünster, Austria). Tras la extracción, se centrifugaron las muestras a 2000 rpm durante 10 minutos para procesarlas inmediatamente y/o se congelaron a −20 °C hasta su análisis. La hs-TnT y la PCR se midieron inmediatamente en un analizador Cobas 6000 (Roche Diagnostics International, Rotkreuz, Suiza) empleando un método electroquimioluminiscente y uno inmunoturbidimétrico, respectivamente. La IL-6 y la PCT se midieron en el plasma almacenado en un analizador Cobas 6000 mediante electroquimioluminiscencia.

Para medir la IPF, se tomaron muestras al ingreso en la UCI y 24 horas después de la cirugía en tubos de 4 mL con ácido tripotásico etilendiaminotetraacético (Vaccuette^®^ , Greiner Bio One^®^, Kremsmünster, Austria) y conservadas a temperatura ambiente hasta su análisis. Todas las muestras se analizaron a las 2–4 h tras su obtención con el sistema Sysmex^®^ XN (Sysmex^®^, Kobe, Japón) siguiendo las recomendaciones del fabricante.

Además, se recogieron los resultados preoperatorios y postoperatorios de magnitudes relacionadas con la función renal y hepática (creatinina, bilirrubina total y alanina aminotransferasa [ALT]), obtenidas con el analizador Cobas 6000 mediante espectrofotometría. Las concentraciones de hemoglobina, leucocitos y plaquetas se midieron con el analizador Sysmex XN. La hemoglobina se midió aplicando el método del lauril sulfato de sodio fotométrico, mientras que los leucocitos y plaquetas se midieron mediante citometría de flujo con fluorescencia con un láser de diodo semiconductor.

Se calculó la puntuación en el sistema de valoración de la gravedad APACHE II en cada paciente en las primeras 24 h de ingreso en la UCI.

### Análisis estadístico

Las variables continuas se presentan como valores medios y de desviación estándar (DE) o como medianas o rangos intercuartílicos (IRC), según corresponda. Las diferencias entre medias entre grupos se analizaron mediante el test t de Student para los datos con distribución normal, o el test U de Mann–Whitney para las variables con distribución asimétrica. Para las variables categóricas, se empleó el test de X^2^ de Pearson o el test de Fisher, según correspondiera.

Se realizó un modelo de regresión logística para evaluar la relación entre los biomarcadores (hs-TnT, PCR, IL-6, PCT, and IPF) y el desarrollo de MACE (variable dependiente).

Los factores de riesgo como la edad, sexo, transfusión intraoperatoria y el tiempo de pinzamiento aórtico se consideraron como potenciales covariantes para el modelo. Únicamente las variables con diferencias estadísticamente significativas se incluyeron en el modelo final. Una probabilidad de ≤0.05 se consideró estadísticamente significativa. Los análisis estadísticos se realizaron con el programa Stata/MP, versión 14 (StataCorp LP).

## Resultados

Durante el periodo del estudio, se incluyeron un total de 210 pacientes (n=210). La Tabla 1 muestra los datos demográficos y preoperatorios de todos los pacientes atendiendo a la presencia o no de MACE.

**Tabla 1:** Características basales de todos los pacientes y subgrupos.

Características	Total (n=210)	Sin MACE (n=179)	MACE (n=31)	Valor p
Sexo masculino, n (%)	137 (65)	120 (67)	17 (57,8)	0,188
Edad, años (min-max)	70 (28–86)	70 (28–86)	71 (72–85)	0,566
IMC, kg/m^2^, media ± DE	30 ± 4,8	29 ± 4,9	27,6 ± 3,5	0,120
**Magnitudes de laboratorio**				
Hemoglobina, g/L, media ± DE	132 ± 16	132 ± 19	131 ± 18	0,847
Leucocitos, ×109, media ± de	8,0 ± 2,3	8,1 ± 2,3	7,4 ± 1,9	0,173
No. de plaquetas, ×109/L, media ± DE	218 ± 63	222 ± 63	188 ± 52	0,009
Creatinina, µmol/L, media ± DE	94 ± 31	94 ± 32	95 ± 25	0,413
Bilirrubina total, µmol/L, media ± DE	10 ± 6	9,7 ± 5,2	12,7 ± 10,9	0,332
ALT, U/L, mediana, IQR	18,6 (13,8–25,8)	18,6 (13,8–25,2)	20,4 (15–28,8)	0,447
**Comorbilidad y tratamiento farmacológico**				
Diabetes mellitus, n (%)	85 (40,5)	73 (40,8)	12 (38,7)	0,828
Hipertensión, n (%)	165 (78,6)	141 (78,8)	24 (77,4)	0,866
Dislipidemia, n (%)	138 (65,7)	119 (66,5)	19 (61,3)	0,574
EPOC, n (%)	19 (9,5)	15 (8,4)	4 (12,9)	0,418
Case III y IV de NYHA, n (%)	73 (34,8)	58 (32,4)	15 (48,4)	0,198
Im previo, n (%)	61 (29)	49 (27,4)	12 (38,7)	0,199
Ictus previo, n (%)	22 (10,5)	16 (8,9)	6 (19,4)	0,080
Aspirina, n (%)	104 (49,5)	90 (50,3)	14 (45,2)	0,599
Beta-bloqueante, n (%)	112 (53,3)	95 (53,1)	17 (54,8)	0,856
Estatinas, n (%)	134 (63,8)	114 (64)	20 (64,5)	0,960

Los datos se expresan en valores numéricos (%) o ,media ± DE o medianas (IQR) según la distribución de los datos. La edad en años se presentan como valores medios (mínimo-máximo). IMC, índice de masa corporal; ALT, alanina aminotransferasa; EPOC, enfermedad pulmonar obstructiva crónica; NYHA, New York heart association; IM, infarto de miocardio.

La mediana de la duración del ingreso en la UCI fue de 3 (2–5) días, y la puntuación APACHE II media fue 12.9 ± 4.6. Un total de 31 (14.8%) pacientes desarrollaron algún evento adverso mayor (Tabla 2). Cinco pacientes presentaron más de un evento. Dos pacientes sufrieron un fallo cardíaco y fallecieron. Un paciente presentó fallo cardíaco e infarto de miocardio, mientras que otro sufrió fallo cardíaco e e ictus. Finalmente, el último paciente tuvo fallo cardíaco, e infarto de miocardio, siendo éxitus finalmente.

**Tabla 2:** Eventos ocurridos tras la CC.

	n (%)
IM, n (%)	6 (2,9)
Ictus, n (%)	2 (0,95)
FC, n (%)	22 (10,5)
Mortalidad a 1-mes, n (%)	7 (3,3)
Al menos un evento cardiovascular adverso mayor (MACE), n (%)	31 (14,8)

Los datos se expresan en cifras (%). MI, infarto de miocardio; FC, fallo cardíaco.

wEl tipo de intervención más frecuente fue la cirugía de una sola válvula (n=83; 38%), seguida de CABG (n=72; 34%), procedimientos combinados (CABG más válvula: n=22; 10.5%), doble válvula (n=17; 8%), cirugía aórtica (n=11; 5.3%), y miscelánea, lo que incluyó principalmente la extracción de un mixoma (n=5; 2.4%). El resto de parámetros quirúrgicos se muestran en la Tabla 3.

**Tabla 3:** Datos quirúrgicos de la totalidad de los pacientes y de los pacientes que desarrollaron y no desarrollaron MACE.

Parámetros quirúrgicos	Total (n=210)	Sin MACE (n=179)	MACE (n=31)	Valor p
Procedimiento quirúrgico, n (%)				0,075
CABG	72 (34,2)	64 (35,8)	8 (25,8)	
Cirugía valvular	83 (39,5)	74 (41,3)	9 (29)	
Doble válvula	17 (8,1)	13 (7,3)	4 (12,9)	
Procedimientos mixtos	22 (10,5)	15 (8,4)	7 (22,6)	
Cirugía aórtica	11 (5,2)	8 (4,5)	3 (9,7)	
Miscelánea	5 (2,4)	5 (2,8)	0	
Cirugía programada, n (%)	155 (73,8)	135 (75,4)	20 (64,5)	0,184
Duración de la intervención, min, media ± DE	277 ± 88	272,5 ± 84	305,9 ± 106	0,085
Duración de CPB, min, media ± DE	103 ± 38	100,6 ± 36	114,3 ± 46	0,115
Tiempo de PAo, min, media ± DE	61,8 ± 37	58,2 ± 36	82,7 ± 36	0,004
Transfusión intraoperatoria de hematíes, n (%)	38 (18,1)	26 (14,5)	12 (38,7)	0,001
Número de concentrados de hematíes, n (%)				0,008
1	20 (9,5)	14 (7,8)	6 (19,4)	
2	15 (7,1)	9 (5)	6 (19,4)	
3	2 (1)	2 (1,1)	0	
4	1 (0,5)	1 (0,06)	0	
Transfusión intraoperatoria de plaquetas, n (%)	24 (11,4)	15 (8,4)	9 (29)	0,001
Número de *pool* de plaquetas, n (%)				<0,001
1	19 (9,1)	14 (7,8)	5 (16,1)	
2	5 (2,4)	1 (0,6)	4 (12,9)	

Los datos se expresan en porcentajes (%), media ± DE. CABG, cirugía de revascularización coronaria; CPB, bypass cardiovascular; PAo, pinzamiento aórtico.

### Biomarcadores

Los biomarcadores (hs-TnT, PCR, IL-6, PCT, and IPF) se midieron inmediatamente tras el ingreso en la UCI y 24 horas tras la CC. La IL-6 y la PCT a las 24 horas se midieron únicamente en 137 (65.2%) de los pacientes debido a la falta de separación del plasma o de refrigeración inmediata tras la extracción, lo cual puede afectar a la estabilidad de la IL-6 [25]. El resto de biomarcadores se midieron de forma convencional dos veces. Se calculó el valor delta de cada biomarcador en todos los pacientes. Este se definió como la diferencia entre el primer día postoperatorio y el día de ingreso en la UCI. Para la IPF (%), la diferencia se expresa en puntos porcentuales (pp).

La dinámica de los biomarcadores mostró valores más elevados en los pacientes con MACE, en comparación con aquellos que no desarrollaron MACE. No obstante, no todos los biomarcardores fueron significativamente mayores en los pacientes que desarrollaron MACE frente a los que no (Tabla 4). Únicamente el valor delta de PCT, hs-TnT, e IPF fueron estadísticamente superiores en el grupo MACE. El cambio porcentual relativo de cada biomarcador fue de 661% en PCT, −3,8% en hs-TnT, y 18% en IPF en el grupo no-MACE y 2.975%, 48%, and 37,5%, respectivamente, en el grupo MACE. Dado que la presencia de infección podría elevar las concentraciones de algunos biomarcadores, principalmente de PCR, IL-6, PCT e IPF, los datos se analizaron excluyendo aquellos pacientes que presentaron infección en los primeros tres días tras la CC (Tabla 4). En total, 13 pacientes desarrollaron infección. El tipo de infección más frecuente fue la neumonía (n=10; 79,9%), seguida de la mediastinitis (n=1; 7,7%), infección de la herida quirúrgica (n=1, 7,7%), e infección del tracto urinario (n=1; 7,7%). Además, el resto de resultados analíticos relacionados con parámetros renales, hepáticos y del hemograma se muestran en la Tabla 5.

**Tabla 4:** Concentraciones de biomarcadores en los pacientes con y sin MACE.

	Tiempo	Todos los pacientes					Sin incluir a los que desarrollaron infección temprana				
		n	Total de pacientes	Sin MACE^a^	MACE^b^	Valor p	n	Total de pacientes	Sin MACE^c^	MACE^d^	Valor p
PCT, mediana (IQR) µg/L	0	210	0,057 (0,037–0,09)	0,056 (0,034–0,09)	0,08 (0,046–0,16)	0,025	197	0,056 (0,035–0,09)	0,055 (0,034–0,090)	0,08 (0,045–0,101)	0,064
	24	137	0,501(0,233–1,68)	0,425 (0,19–1,09)	2,46 (0,67–5,34)	<0,001	128	0,43 (0,216–1,635)	0,415 (0,186–1,08)	2,175 (0,69–4,64)	0,002
Delta de PCT µg/L		137	0,414 (0,149–1,547)	0,373 (0,136–1,01)	2,054 (0,624–5,25)	<0,001	128	0,387 (0,141–1,409)	0,362 (0,131–0,955)	1,937 (0,645–4,60)	0,001
IL-6, mediana (IQR) ng/L	0	210	183,2 (112,9–315)	177,5 (108,7–282,9)	228,8 (153,5–621)	0,010	197	181,3 (111,8–291,8)	174,4 (106,2–282,75)	218,7 (159–404,5)	0,032
	24	137	160,2 (104,3–241,5)	156,1 (102,2–228,1)	241,5 (107,2–508,6)	0,057	128	156,1 (103–234,2)	156,1 (102,2–227,3)	161,1 (104,3–421,4)	0,502
Delta de IL-6 ng/L		137	23,2 (−158,6–59,7)	−5,53 (−142,4–59,7)	−87,4 (−382,6–172,8)	0,462	128	−15,3 (−142,6–59,7)	−5,5 (−130,4–59,7)	−64,4 (−215,4–51,5)	0,614
PCR, mediana (IQR) mg/L	0	210	2,3 (1–4,5)	2,3 (1–4,6)	2,5 (0,8–4,1)	0,998	197	2,3 (1–4,5)	2,3 (1–4,5)	2,2 (0,8–4,1)	0,802
	24	210	148,4 (103,4–201)	146,1 (103,4–196,5)	167,6 (97,5–227,6)	0,348	197	149,1 (96,4–199,6)	147,4 (97,5–196,1)	164,4 (88,5–225,9)	0,542
Delta de PCR mg/L		210	145,3 (93,8–199)	142,4 (94,7–192)	163 (90,3–223)	0,5	197	145,6 (92,3–200,8)	142,4 (92,9–192,1)	172,9 (80,4–222,5)	0,057
Hs-TnT, mediana (IQR) ng/L	0	210	415 (250,5–795)	390 (241–708)	626 (387–1631)	0,001	197	400 (249–730)	387 (240–706)	576 (369–1344)	0,013
	24	210	409 (265–719)	375 (249–579)	926 (490–1580)	<0,001	197	388 (255–674)	366 (246–374)	828 (423–1244)	<0,001
Delta de hs-Tnt ng/L		210	−14,8 (-246-133)	−31,9 (-248-72)	139 (−45,5–583)	0,003	197	−18,8 (−246,3–115)	−35,4 (-249-72)	120 (-6–461)	0,006
IPF, mediana (IQR) %	0	210	4 (2,6–5,4)	3,9 (2,6–5,4)	4 (2,7–5,4)	0,547	197	3,9 (2,6–5,3)	3,9 (2,6–5,4)	4 (3,2–5,3)	0,696
	24	210	4,7 (3,2–6,6)	4,6 (3,1–6,5)	5,5 (4,1–9,1)	0,025	197	4,6 (3,1–6,5)	4,6 (3–6,5)	5,3 (4,1–7,1)	0,151
Delta de IPF pp		210	0,7 (0–1.5)	0,6 (0–1,4)	1 (0,1–2,5)	0,048	197	0,5 (0–1,3)	0,5 (−0,1-1,3)	1 (0,1-1,9)	0,014

Los valores se expresan en medianas (IQR). PCT, procalcitonina; IL-6, interleucina-6; PCR, proteína C-reactiva; hs-TnT, troponina T de alta sensibilidad; IPF, fracción de plaquetas inmaduras; PP, puntos porcentuales. ^a^n=179, ^b^n=31, ^c^n=172, ^d^n=25. 24 h después de la CC para PCT, IL-6 y sus valores delta: ^a^n=118, ^b^n=19, ^c^n=114, ^d^n=14.

**Tabla 5:** Resultados analíticos postoperatorios.

	Total (n=210)	Sin MACE (n=179)	MACE (n=31)	Valor p
**Postoperatorio (ingreso en la UCI)**				
Hemoglobina, g/L, media ± DE	106 ± 15	107 ± 15	101 ± 15	0,129
Leucocitos, ×10^9^/L, media ± DE	13,1 ± 5,5	13,0 ± 5,6	13,0 ± 4,6	0,590
Plaquetas, ×10^9^/L, media ± DE	164 ± 57	168 ± 58	143 ± 46	0,022
Creatinina, µmol/L, media ± DE	79 ± 32	79 ± 34	81 ± 23	0,250
Bilirrubina total, µmol/L, media ± DE	15 ± 9	15 ± 8	19 ± 11	0,038
AL, U/L, mediana, IQR	17 (13–25)	16 (13–24)	20 (14–31)	0,066
**Postoperatorio (24 tras la cc)**				
Hemoglobina, g/L, media ± DE	101 ± 14	101 ± 13	98 ± 16	0,405
Leucocitos, ×10^9^/L, media ± DE	14,4 ± 4,3	14,1 ± 4,1	16,3 ± 5,1	0,019
N° de plaquetas, ×10^9^/L, media ± DE	166 ± 54	169 ± 56	148 ± 48	0,073
Creatinina, µmol/L, media ± DE	90 ± 50	87 ± 47	112 ± 61	0,004
Bilirrubina total, µmol/L, media ± DE	13 ± 9	12 ± 9	17 ± 14	0,013
ALT, U/L, mediana, IQR	19 (15–29)	18(14–25)	34 (22–102)	<0,001

Los datos están expresados en porcentajes (%) o valores medios ± DE o mediana (IQR) según la distribución de los datos. ALT, alanina aminotransferasa.

### Regresión logística

El análisis univariado mostró diferencias entre los pacientes con y sin MACE en lo relativo al tiempo de PAo, transfusión de hemoderivados, el valor delta de la troponina y el valor delta de IPF. Por otro lado, no hubo diferencias relacionadas con la edad o el sexo (Tabla 6). Tras el ajuste por tiempo de PAo y transfusión de hemoderivados, la relación entre el valor delta de IPF y el valor delta de hs-TnT con el desarrollo de MACE siguió siendo estadísticamente significativa. El valor delta de IPF fue el que mostró una mayor relación independiente, con una OR de 1,47 (IC: 1,11–1,96). Por otro lado, el valor delta de hs-TnT también mostró una relación independiente, con una OR de 1,001 (IC: 1,0002–1,001). Es decir, con cada aumento de 100 ng/L en el valor delta de hs-TnT, el riesgo de presentar un MACE aumentaba en un 10% (Tabla 6).

**Tabla 6:** Modelo de regresión logística de variables asociadas al desarrollo de MACE.

	Univariante		Multivariante	
Variables	OR (IC 95%)	Valor p	OR (IC95%)	Valor p
Edad	1,02 (0,98–1,06)	0,380	–	–
Tiempo de PAo	1,02 (1,01-1,03)	0,001	1,02 (1,01-1,03)	0,003
Transfusión de hemoderivados	2,77 (1,45–5,32)	0,002	1,97 (0,90–4,28)	0,089
ΔIPF	1,55 (1,17–2,07)	0,002	1,47 (1,11–1,96)	0,008
ΔPCT	1,10 (0,97–1,24)	0,124	–	–
Δhs-TnT	1,001 (1,0002–1,002)	0,010	1,001 (1,0002–1,001)	0,008

OR, odds ratio (razón de probabilidades); IC, intervalo de confianza; tiempo de Pao, tiempo de pinzamiento aórtico; ΔIPF, valor delta de la fracción de plaquetas inmaduras; ΔPCT, valor delta de procalcitonina; Δhs-TnT, valor delta de troponina T de alta sensibilidad.

### Discusión

A pesar de los avances y mejoras en la atención de los pacientes de cirugía cardíaca, las complicaciones cardiovasculares tras CC siguen representando un riesgo importante que puede afectar los resultados clínicos de los pacientes. En este estudio prospectivo realizado en más de 200 pacientes que se sometieron a cirugía cardíaca, se halló una relación entre el valor delta de la fracción de plaquetas inmaduras y el valor delta de la troponina T de alta sensibilidad (en menor medida), y el desarrollo de MACE en el postoperatorio, independientemente de la presencia de factores de riesgo.

Los resultados de este estudio sugieren que IPF y hs-TnT podrían ser marcadores útiles a la hora de evaluar el riesgo de desarrollar MACE tras CC. Este resultado coincide con los publicados en otros estudios. Numerosos estudios han mostrado que la hs-TnI/T está relacionada con MACE [26], [27], [28]. Hasta la fecha, solo un estudio ha señalado la relación entre la IPF y MACE, aunque dicho estudio se realizó en otro tipo de pacientes que no se habían sometido a cirugía cardíaca [5]. No existen estudios previos en los que se haya demostrado esta relación en pacientes de CC.

La CC puede empeorar o aumentar la respuesta inflamatoria, lo cual tiene importantes implicaciones clínicas, que pueden derivar en un fallo cardíaco. Existen numerosos mecanismos bioquímicos y moleculares implicados en la patogénesis de la respuesta inflamatoria: la alteración en la generación de óxido nítrico, el aumento de las concentraciones de citocinas proinflamatorias y la disfunción del endotelio, entre otros. En consecuencia, la respuesta inflamatoria tras la CC puede incrementar el riesgo de que se produzca un evento cardíaco postoperatorio, así como causar disfunción orgánica [29].

Dado que la respuesta inflamatoria podría estar implicada en la patogénesis de eventos cardiovasculares tras la CC, planteamos la hipótesis de que los biomarcadores inflamatorios como la PCT, PCR, IL-6, y la IPF podrían estar relacionados con el desarrollo de MACE.

Tal como esperábamos, descubrimos que algunos parámetros analíticos relacionados con la función renal y hepática, así como los leucocitos eran más elevados durante el postoperatorio en los pacientes que desarrollaron MACE frente a los que no. El desarrollo de MACE están estrechamente relacionados con la respuesta inflamatoria. Del mismo modo, la respuesta inflamatoria contribuye a la patogénesis del fallo orgánico [29].

Por otro lado, en nuestro estudio, observamos que 31 pacientes cumplieron al menos uno de los criterios de MACE. No se hallaron diferencias entre el tipo de intervención y el desarrollo de MACE. Con respecto a los biomarcadores, todos estaban elevados en el grupo MACE, con diferencias estadísticamente significativas en los valores delta de PCT, hs-TnT, e IPF. No se encontraron diferencias en los valores del delta de IL-6 y PCR. Está ampliamente aceptado que la PCT, IL-6, PCR y la IPF están estrechamente relacionadas con las infecciones. También analizamos los resultados tras excluir a los pacientes que desarrollaron infección en los primeros tres días del postoperatorio.

El comportamiento de los valores delta de los diferentes biomarcadores fue el mismo incluso tras excluir a los pacientes con infección en el postoperatorio inmediato. Con respecto a la IL-6, un estudio reciente en pacientes adultos sometidos a CC mostró una relación estadísticamente significativa entre las concentraciones de IL-6 y la mortalidad tras la CC [30]. A diferencia de nuestro estudio, en aquel se analizó la mortalidad a un año.

Aunque las diferencias en los valores del delta de PCR no fueron estadísticamente significativas, su comportamiento tendía a ser el mismo (positivo) en ambos grupos. Las concentraciones de PCR en suero fueron inferiores en el momento del ingreso en la UCI, comparados con las de PCR 24 horas tras la cirugía, lo cual puede estar relacionado con la cinética de esta proteína de fase aguda. Las concentraciones de PCR en el postoperatorio inmediato empiezan a incrementarse a las 2 horas, alcanzando su valor máximo tras 48 horas [31].

La cirugía mayor comúnmente desencadena una notable respuesta inflamatoria, la PCR aumenta tras la CC como respuesta a la inflamación inducida de forma inespecífica por el trauma quirúrgico. Este hecho podría explicar la ausencia de diferencias entre las medias de PCR en ambos grupos de pacientes. Por tanto, la PCR no resulta útil en este contexto, ya que también suele estar elevada en pacientes con una evolución favorable.

Algunos estudios han mostrado concentraciones más elevadas de PCT en los pacientes con complicaciones tras la CC. El estudio llevado a cabo por Liu et al. [32] muestra un pico de PCT en las 24 primeras h del postoperatorio, observando concentraciones significativamente mayores de PCT, así como de aclaramiento de PCT, en el grupo de fallecidos, en comparación con los supervivientes de CC. Clementi et al. [13] observaron que las concentraciones de PCT 48 h después de la CC fueron significativamente mayores en pacientes con eventos renales y respiratorios o que desarrollaron sepsis. En concordancia con nuestro estudio, los autores no hallaron diferencias en los resultados cardiovasculares entre los pacientes que fallecieron durante el ingreso, a los 30 días o a los 6 meses.

La troponina T o I son biomarcadores cardíacos que reflejan el mecanismo fisiopatológico de MACE desde el punto de vista del daño miocárdico. En un estudio llevado a cabo con una amplia cohorte de pacientes con enfermedad coronaria, concentraciones elevadas de hs-TnI se relacionaron con un mayor riesgo de MACE [8]. Mauermann et al. [26] analizaron la relación entre las variaciones en la troponina entre el primer y segundo día del postoperatorio en pacientes de CC con mortalidad por cualquier causa y la morbilidad cardíaca. Así, los autores hallaron que un incremento de más del 10% en las concentraciones de troponina, estaba significativamente relacionado con el pronóstico de los pacientes. Todos estos hallazgos respaldan los resultados obtenidos en este estudio, ya que la asociación estadísticamente significativa se mantuvo incluso tras el ajuste por el tiempo de pinzamiento aórtico en el análisis multivariado.

Los últimos estudios sobre IPF se han centrado en su posible relación con los resultados clínicos en pacientes con enfermedades cardiovasculares que se han sometido a cirugía mayor. Aunque se sabe que la producción de IPF está inducida por las bajas concentraciones de plaquetas en sangre, su papel en condiciones inflamatorias no es tan conocido, y el mecanismo por el cual los niveles de IPF aumentan aún no ha sido investigado. Existe cada vez mayor evidencia de que el mecanismo fisiopatológico mediante el cual la IPF aumenta tras un estímulo inflamatorio está relacionado con la regulación de la trombopoyetina en respuesta a los mediadores de fase aguda. La IL-6 aumenta la transcripción de la trombopoyetina en el hígado y, como consecuencia, la trombopoyetina aumenta provocando una reacción en la producción de plaquetas [33].

Algunos estudios han demostrado la asociación entre la IPF y las complicaciones en la respuesta inflamatoria, aunque solo uno de ellos se realizó en pacientes sometidos a CC [17]. La IPF podría ser un biomarcador de utilidad para la identificación temprana de aquellos pacientes con mayor riesgo de desarrollar una respuesta inflamatoria tras la CC. Existen numerosos estudios sobre el papel que juega la IPF en el desarrollo de enfermedades infecciosas. Algunos de ellos han demostrado su utilidad, especialmente en los pacientes con sepsis. Park et al. [34] observaron que la IPF tiene una elevada sensibilidad y especificidad a la hora de discriminar a los pacientes con sepsis de aquellos que no la presentan. Además, Koyama et al. [35] hallaron que los niveles de plaquetas inmaduras están asociados a una mayor mortalidad en los pacientes con sepsis. Sin embargo, la bibliografía sobre la relación entre la IPF y las complicaciones postoperatorias, es aún escasa. Nuestros datos coinciden con los del estudio realizado por Anetsberger et al. [5]. Estos autores observaron que la FPI en el postoperatorio está relacionada con un mayor riesgo de desarrollar un evento cardiovascular adverso mayor, así como un tromboembolismo, en pacientes que se han sometido a CC.

### Limitaciones del estudio

La principal limitación de este estudio es el pequeño tamaño muestral. El tamaño de la muestra ha limitado el número de variables a analizar para determinar la relación independiente de los biomarcadores con el desarrollo de MACE. En segundo lugar, en un elevado número de pacientes, no pudimos determinar la concentración de la PCT y la IL-6 a las 24 horas después de la cirugía, debido a la baja estabilidad de la IL-6. Por último, en el postoperatorio solo se midieron dos biomarcadores (al ingreso en la UCI y 24 h después de la cirugía). Sin embargo, pensamos que el valor delta entre estos dos momentos podría proporcionar información fiable y de forma más temprana que otros intervalos, como, por ejemplo, el ingreso en la UCI y 48 horas después de la CC. Finalmente, no se midió la IPF en el preoperatorio. No obstante, en un estudio previo realizado en pacientes sometidos a cirugía no cardíaca en el que se investigó la relación entre la IPF y MACE, no se observaron diferencias significativas de este biomarcador entre los grupos [5].

## Conclusiones

En conclusión, valores elevados del delta de IPF y hs-TnT entre la hora 0 y las 24 horas posteriores a la cirugía cardíaca mostraron estar relacionadas con peores resultados clínicos, en términos de eventos cardiovasculares adversos durante el ingreso en la UCI y la mortalidad a los 30 días. Este estudio demuestra que los valores delta de la IPF y hs-TnT siguen asociándose al desarrollo de MACE incluso tras ajustar el modelo con factores intraoperatorios, como el tiempo de pinzamiento aórtico y la transfusión de hemoderivados. Nuestros datos sugieren que medir la IPF y la troponina en el postoperatorio sería de utilidad a la hora de evaluar el riesgo de MACE en los pacientes de CC.

Estos datos presentan un potencial interés, ya que la IPF es una magnitud que se puede incluir fácilmente en los análisis en la práctica clínica habitual a un bajo coste.
